# Open Maxillary Sinus with Concomitant Maxillary Sinusitis

**Published:** 1899-08

**Authors:** Faneuil D. Weisse


ARTICLE II.

### OPEN MAXILLARY SINUS WITH CONCOMITANT MAXILLARY SINUSITIS.

FANEUIL D. WEISSE, M. D.

Read before the First District Dental Society of the State of New York, December 13th, 1898.

In order to have a proper conception of this condition I propose to preface the direct consideration of the subject with some brief practical remarks on the anatomy and physiology of the maxillary sinus, together with a review of the causes which lead to the development and perpetuation of open maxillary sinus with concomitant maxillary sinusitis. I trust that I may not be regarded as too iconoclastic or revolutionary in my views, and that they may commend themselves as founded on anatomical, physiological and pathological conditions; the therapeutical indications to be fulfilled and the means employed in carrying out the same have been proven by repeated experience in practice.

*Anatomy.*—The sinus maxillaris, maxillary sinus, antrum, and antrum of Highmore are the several names given to the cavity in the body of the superior maxillary bone. (Nathaniel Highmore, an English physician, born 1613, died 1684, is usually thought to have been the first who described this cavity. This is an error, as it had been described by earlier anatomists, Galen described it under the name of "sinus maxillare." All English writers since Highmore have designated it as the "antrum of Highmore)." The proper anatomical name for this cavity is *sinus maxillaris*, or maxillary sinus, thus associating it, as it should be, with the other diverticula from the nasal cavity. The names "antrum" and "antrum of Highmore" should be discarded. These sinuses, like those in the body of the sphenoid and the frontal bones and the cells of the lateral masses of the ethmoid bone, are cavities hollowed out of the bone structure and lined by periosteum and mucous membrane continued into them from the nasal cavities.

The walls of a maxillary sinus form the body of a superior maxillary bone, which has the shape of a vertical wedge; the apex of the wedge is at the malar articulation of the bone; the base forms the outer wall of the nose cavity; the superior face forms the floor of the orbit; the exterior face forms the internal wall of the zygomatic fossa; the anterior face forms the facial surface; the inferior face —forming the floor of the sinus—has developed upon it the alveolar process of the bone. It is to be noted here that the sinus has its floor structure proper before the development of the scaffolding structure of the alveolar process for the lodgment of the roots of the teeth, and after the absorption of the alveolar process consecutive to the loss of the teeth. In illustration of this I show you these two skulls; one of about the period of birth, and the other at the period of old age, after the teeth have been shed; in both specimens a perfect floor or inferior wall of the maxillary sinus presents.

The roots of the teeth of the upper arch are seemingly projected as if to perforate the floor of the sinus, but in reality the alveolar process develops so as to socket the entire roots of the teeth exteriorly to the proper osseous floor of the sinus. At times the encroachment of certain teeth, usually the second and first permanent molars, is such as to raise the floor of the sinus into elevation. In these cases, always the alveoli are lined by the tissues at the apical spaces at the roots of the teeth, while the sinus, opposite to the alveolus, is lined by a portion of the periosteum and mucous membrane common to the entire sinus cavity. It is stated by some authorities that the floor of the sinus is at times perforated by the roots of the second or first molar teeth, but these instances are probably from post-mortem specimens or after pathological conditions had existed in the alveolaris of the root involved. In post-mortem specimens presenting perforation, the perforations are due to the handling in the cleaning and the process of maceration of the bone; while in pathological specimens perforations are due to destruction by necrosis of the osseous floor of the sinus. In this skull you will see an illustration of the usual perforation—a round hole found in the inferior wall of the maxillary sinus, caused in all probability by the process of maceration of the skull.

The roots of the teeth—cuspids, bicuspids and molars—as socketed in the alveolar process, the osseous scaffolding developed for their lodgment inferiorly to the special osseous flooring of the sinus, have varying relations to the cavity of the sinus, that is, their roots vary in the nearness of their approach to the cavity of the sinus. In this specimen the upper cuspid, bicuspids and molars of a side have been extracted; the labial walls of the alveoli have been broken away, and the interior wall of the sinus has been broken away, so as to expose the interior of the floor of the cavity of the sinus. This preparation thus enables us to appreciate the nearness of approach of the roots of these teeth to the cavity of the sinus. The cuspid alveolus runs up into the root of the nasal process of the superior maxilliary bone, entirely internal to the cavity of the sinus and separated by osseous tissue from it. The first and second bicuspid alveoli, less deep, and lodged entirely in alveolar process, are well separated by osseous tissue from the sinus cavity. The first molar alveolus, bedded in alveolar process, approaches closer to the sinus cavity, but it is separated from it by a well-defined thickness of osseous tissue. The second molar alveolus, lodged in alveolar process, approaches much closer than any of the others to the sinus cavity, being separated from it by only a thin layer of osseous tissue. The third molar alveolus, lodged in alveolar process posteriorly to the sinus cavity, is well separated by osseous tissue from the cavity of the sinus. From the specimen, the order of the nearness of approach of the roots of these teeth, as lodged in the alveolar process, to the cavity of the maxillary sinus is as follows: Second molar, first molar, second bicuspid, third molar, first bicuspid and cuspid.

It is stated by embryologists that the maxillary sinus is present as early as the sixth week of fetal life; thereafter it increases in size with the development of the bone. In adults it is larger in males than in females, but it is subject to many variations in size, even in the same subject on the two sides. The base wall of the body of the bone, which is also the base of the sinus cavity, forming the outer wall of the nose cavity, presents a large deficiency, which is greatly diminished by the application to the internal face of the body of the bone of portions of the palate, ethmoid, and turbinate bones. The comparatively small aperture remaining, located in the middle meatus area of the nose cavity, is the point of communication of the sinus with the nose cavity. The posterior, external, superior, and anterior walls of the sinus are tunneled by canals for the transit of vessels and nerves to and vessels from the teeth, the mucus membrane of the maxillary sinus, and the gums. The exterior of the body of the bone is covered by periosteum and the superimposed parts. The interior of the maxillary sinus is lined by a delicate and peculiarly structured periosteum, which in turn is lined by a closely adherent mucus membrane; the later is rich in blood-vessels, lymphatic vessels, and nerves, and particularly rich in mucous glands of large size and varying shapes. With some modification, the periosteum and the mucous membrane of the maxillary sinus are identical with those of the nasal cavity and its other diverticula. The osseous borders of the aperture of communication between the maxillary sinus and the nose cavity are covered by periosteum and mucous membrane in transit from the nose cavity into the sinae, and their presence narrows the opening to the size of admiting a probe; a quill, or even a larger body. The circumferential mucous membrane at this opening is to be remembered, as in some cases of maxillary sinusitis its swelling may obstruct the flow of pus from the sinus into the nasal cavity.

*Physiology.*—The functions of the maxillary sinus are:

1. Respiratory, in common with the other diverticula of the nose cavity, in that they are chambers for the warming of the air. Air, always being present in them, is warmed by contact with their interior mucous membrane, which contains capillaries filled with blood at 98½° F., which warmed air is drawn down by inspiration into the larynx, trachea, etc.

2. Vocal, as reverberating cavities for the modulation of the voice sounds.

*Pathology.*—Diseases of the maxillary sinus may be divided into:

1. Primary or initial (not under consideration in this article), due to pathological changes of the linings or walls of the sinus.

2. Secondary or sequential, due to cases exterior to the sinus.

The secondary or sequential diseases of the maxillary sinuses are:

1. Empyema.
2. Maxillary sinusitis.

1. *Empyema of the maxillary sinus* is the correct pathological name to apply to the presence of pus in this cavity. (I have always maintained that the term "abscess" as applied to the maxillary sinus is a pathological misnomer. The pus of an abscess, resulting as it does from inflammation of the central tissue of a part, is lodged within tissue, while in the case of pus in the maxillary sinus the pus is in an actual cavity. I was much pleased in reading the following in Mr. Salter's article in Holme's "System of Surgery:" "The term 'abscess of the antrum' conveys a wrong impression of the real nature of this disease; it is not the suppuration of inflamed parenchyma, but the occlusion in a cavity of the purulent secretion from the surface of a mucous membrane which lines that cavity)." This condition may be due to the following causes:

*a.* The entrance into the sinus of pus from an abscess of a contiguous alveolus or from a contiguous sinus, or from the nose cavity.

*b.* The presence of pus in the sinus from maxillary sinusitis.

2. *Maxilliary sinusitis* is a name which I would give to exudative inflammation of the mucous membrane lining the sinus. The causes of maxillary sinusitis are:

*a.* Acute and chronic rhinitis.

*b.* Foreign bodies in the sinus.

*c.* Traumatism.

*d.* Inflammation or abscess in contiguous alveolar cavities, with consecutive abnormal opening into the sinus.

*a.* Acute and chronic rhinitis are, because of the continuity of the mucous membrane from the nose cavity into the sinus, self-evident causes.

*b.* Foreign bodies in the sinus are most often the result of accidents in the course of the, at present, usual treatment of open maxillary sinus.

*c.* Traumatism is a cause at all ages. Cases in infants a week after birth have been reported where maxillary sinusitis had been induced by trauma, due to forceps delivery.

*d.* That morbid conditions of the teeth—molars, bicuspids, and cuspids—and their alveoli are the most frequent exterior causes of open maxillary sinus with concomitant maxillary sinusitis is almost the universal opinion of surgical authorities, and such has been my experience for the past twenty-five years. In adults, in by far the majority of cases, the following is the history of the prodromata of an open maxillary sinus with concomitant maxillary sinusitis: One of the teeth (most often the second permanent molar) becomes diseased by caries of the tooth extending to exposure of the pulp; periodical attacks of pulpitis follow. If the tooth condition is neglected pericementitis and alveolar abscess develop; the abscess may open into the sinus, inducing empyema, or it may open at the labial wall of the alveolus. (The contiguous inflammation of the alveolus causes irritation of the periosteum and mucous membrane of the sinus, and a secondary or sequential maxillary sinusitis is induced, which manifests the following symptoms: Deep-seated discomfort or pain in the cheek area; this however is usually referred to the affected tooth, and some pain on pressure over the wall of the sinus. This insidious sinusitis, coincident with the alveolar inflammation, has its focal point at the floor of the sinus, directly opposite the diseased alveolus, and a protective formation of bone occurs at this point, which thickens the osseous floor of the sinus between the alveolus and the sinus cavity. In Dr. Cryer's latest article on the maxillary sinus there is a beautiful illustration of the formation of new osseous tissue at the floor of the sinus in a case of this kind. This thickening of the bone at this point is an attempt to protect the cavity from the intrusion into it of the products of inflammation existing in the alveolus). If extraction of the tooth is delayed, the confined pus, pressing on the walls of the alveolus, interferes with their blood-supply, and its osseous structure undergoes degeneration, by which it is rendered friable. The same change also takes place in the osseous septum between the alveolus and the sinus cavity. Finally the tooth is extraced, and, although the greatest care may be taken in the operation, more or less of the alveolar wall is comminuted, owing to the degeneration of the osseous structure; and after the extraction there .will be found an opening into the maxillary sinus.

As a rule, the opening into the sinus is not at first recognized, attention being called to the condition by the patient's complaining of deep-seated pain in the superior maxillary region; that air and sometimes fluids pass from the mouth to the nose, and at times that there is a discharge of pus from the nasal cavity of the side. There may also be a change in the patient's voice. The symptoms will be attended by persistent non cicatrization of the alveolus, with continued discharge of pus from it. An examination with the probe will, in such cases, determine an abnormal opening into the sinus, and account for the existence of the concomitant maxillary sinusitis. Sometimes an open sinus with concomitant maxillary sinusitis is not preceded by exactly the above prodromata; there may be the following variation: Retained roots of teeth, usually those of the upper second molar, that have been keeping up a chronic pericementitis, with, of course, free drainage at their distal peripheries, which has induced degenerative changes in the osseous walls of the alveolus and the septum between the alveolus and the sinus cavity, with concomitant chronic maxillary sinusitis. These roots being extracted, an open maxillary sinus is present, and there will be a continuance of the maxillary sinusitis.

*Treatment.*—It is not necessary to detail the methods of treatment resorted to—of injections of irritating antiseptics and stimulating solutions into the sinus—or to speak of the usual measures resorted to to keep the opening patent by tents and carefully adapted drainage tubes. With all due respect, I have never resorted to these measures, as I have regarded them as tending to aggravate and to perpetuate the abnormal conditions. Long-standing cases of open maxillary sinus with continuous discharge of pus, after active local treatment, are too often thought to be due to some undiscovered condition of the interior of the sinus—ulceration of its mucous membrane or necrosis of its walls—and heroic measures are resorted to expose its interior by enlarging the alveolar opening or removing its anterior wall or opening the cavity at the inferior meatus of the nose, for the purpose of freer drainage or of curetting interior. All such conditions, if present, I claim will have been due to the treatment, and that they might have been avoided by an early, conservative local treatment, with attention to the systemic condition of the patient.

The local and systemic treatment of a given case of open maxillary sinus with concomitant maxillary sinus with concomitant maxillary sinusitis should be determined upon after having appreciated the following conditions:

1. Determine the exact condition of the sinus by the character and quantity of the discharge from it.

2. Ascertain the existence of any peripheral sources of irritation to the sinus.

3. Determine the existence of any systemic condition likely to impair the recuperative powers of the system.

4. Realize the nature of the changed physiological state of the sinus cavity.

1. The character and quantity of the discharge will indicate the degree of inflammation of the mucous membrane of the sinus. Almost invariably it will be found to be in a septic condition, evidenced by more or less pus flowing from the abnormal opening, and at times from the nose cavity of the side; and hence the perpetuation of the sinusitis. To meet this condition the patient should be given a suitable syringe and instructed how to inject the sinus by the abnormal opening. This he should do at least five times a day with solution of sodii hyposulfitis, one drachm to one ounce of water. As this treatment is carried out the character of the discharge should be noted, as to whether it loses its purulent character and becomes mucus. When tho discharge has become mucus, and has continued so for some time, there will be noticed a gradual closure of the opening, with a change from redness and swelling of the edges of the opening, while the discharge was purulent, to a normal tint of the edges of the orifice. Under the latter conditions the syringe nozzle will not enter the opening as easily, and attempted injection is attended by a certain amount of backflow. Under these circumstances no force should be used to effect injection of the cavity, and as the closure of the opening proceeds the injection should be diminished in frequency, and finally suspended.

2. All existing peripheral sources of irritation should be treated. Remove any roots of teeth present in any of the alveoli of the upper jaw of that side, and keep their alveoli thoroughly asceptic until they are cicatrized. Any teeth in the upper jaw that are dead should be thoroughly treated by removing pulps, cleaning out root-canals, etc. Any rhinitis of the nose cavity of the side should be treated.

3. If any deviation from the normal of any important function exists, the patient should be placed under a regimen of life and remedies calculated to correct the same, so as to bring about a systemic condition of good health. I have improved many cases and put them on the road to a cure by careful attention to the general health. This latter indication is almost invariably neglected.

4. To realize the nature of the changed physiological state of the sinus cavity we should appreciate that it is a secluded cavity, extremely susceptible to irritation, even by slight modifications of its normal condition; that the only access of air to and from it is at the opening by which it communicates with the nose cavity; that normally, air entering and leaving it never passes through it by currents; that its mucous surface is continually bathed by a secretion from the large mucous glands of its mucous membrane, which protects it from irritation from the air entering it. With an abnomal opening into it an abnormal amount of air reaches it, and with the opening at an alveolus each inspiration and expiration is attended by a current of air passing through it from nose to mouth, and the reserve with the mouth open. This abnormal access of air in currents dries the surface of the mucous membrane. This in itself is an irritation to the membrane which tends to perpetuate the maxillary sinusitis. A parallel condition is seen, but to greater degree, where the mucous membrane of the nose cavity is subjected to abnormal access of air, in cases of fissure of the soft and hard palate. In this latter case a chronic rhinitis develops, which induces a permanent induration of the abnormally exposed and dry mucous membrane, thus adapting it to the existing abnormal conditions. Furthermore, with the maxillary sinus open to the buccal cavity, eating and drinking is attended by the ingress of food and drink into the sinus and through it to the nose. These abnormal conditions realized, a most important indication of treatment presents; that of restoring the open maxillary sinus, as nearly as possible, to its normal physiological state of a secluded cavity. To effect this the following measure has been resorted to with success: Close the face of the abnormal aperture by a closely-fitting hard rubber cap, which should overlap upon the labial and palatal surfaces of the gum and be held in place by suitable fixation to the adjacent teeth. This is be to worn during the day, especially when eating and drinking. After each meal it is to be taken out and carefully washed in the hyposulfite solution used for the injections. At night it is to be removed. In the morning, before introducing the cap, after meals, before replacing the cap, and at bed-time, after taking out the cap, are the times when the injections should be made into the sinus.

This cap over the abnormal opening restores the sinus as nearly as possible to its physiological state of a secluded cavity; it prevents undue access of air and currents of air, which cause the mucous membrane lining the cavity to resume its normal moist condition. Irritation removed, the mucous glands resume their secretion. As worn at meals, it effectually prevents the entrance of fluids or particles of food; and it does not interfere with the discharge of pus or mucus, as they readily overflow the cap.

During the past twenty years and more I have treated not a small number of open maxillary sinuses with concomitant maxillary sinuses of varying periods of duration, and after they had undergone manifold methods of treatment and operative procedures. I never have had a case that came to me soon after the abnormal opening was developed, before having undergone any treatment, that I

have failed to arrest the existing maxillary sinusitis and to induce a prompt closure of the abnormal opening. In those that I have not seen early enough, where the opening had been enlarged by operation and the mucous membrane had been subjected to irritation by remedies and the wearing of tents and drainage-tubes, I have succeeded only after prolonged treatment in bringing about a favorable result; and in the cases that had undergone heroic operative treatment I have only been able to render them more comfortable. The experience that I have had warrants me in making a crusade on all irritating local treatment and on all operative measures in cases of open maxillary sinus and concomitant maxillary sinusitis, and in believing that the immediate carrying out of the treatment outlined above will be attended by good results.

The following is an epitome of the treatment to be carried out;

1. Render the sinus aseptic by the mildest possible injections (sodii hyposulfitis one drachm, water one ounce), watching their effect on the discharge (changing from purulent to mucous), and be governed accordingly as to their continuance.

2. Remove all peripheral sources of irritation to the sinus.

3. Place the patient, according to conditions, on a proper regimen of life and course of medication to induce a healthy systemic condition.

4. Restore the sinus as nearly as possible to its physiological condition as a secluded cavity, by a suitable hard rubber cap worn over the abnormal opening.—*Cosmos.*

